# *Hesperomyces* (Fungi, Ascomycota) associated with *Hyperaspis* ladybirds (Coleoptera, Coccinellidae): Rethinking host specificity

**DOI:** 10.3389/ffunb.2022.1040102

**Published:** 2023-01-09

**Authors:** Warre Van Caenegem, Piotr Ceryngier, Jerzy Romanowski, Donald H. Pfister, Danny Haelewaters

**Affiliations:** ^1^ Research Group Mycology, Department of Biology, Ghent University, Ghent, Belgium; ^2^ Institute of Biological Sciences, Cardinal Stefan Wyszyński University, Warsaw, Poland; ^3^ Farlow Reference Library and Herbarium of Cryptogamic Botany, Harvard University, Cambridge, MA, United States; ^4^ Faculty of Science, University of South Bohemia, České Budějovice, Czechia; ^5^ Biology Centre of the Czech Academy of Sciences, Institute of Entomology, České Budějovice, Czechia

**Keywords:** arthropod-associated fungi, Coccinellidae, DNA barcoding, integrative taxonomy, *MCM7*, phylogeny, nuclear ribosomal DNA

## Abstract

Laboulbeniales (Ascomycota, Laboulbeniomycetes) are biotrophic microfungi always attached to the exoskeleton of their arthropod hosts. They do not form hyphae or a mycelium; instead, they undergo determinate growth, developing from a two-celled ascospore to form a multicellular thallus. *Hesperomyces virescens* has been reported on over 30 species of ladybirds (Coleoptera, Coccinellidae); in reality, it represents a complex of species, presumably segregated by host genus association. In this study, we report on *Hesperomyces* thalli on *Hyperaspis vinciguerrae* from the Canary Islands and compare them with the *Hesperomyces hyperaspidis* described on *Hyperaspis* sp. from Trinidad. We generated the sequences of the internal transcribed spacer (ITS) region, the large subunit (LSU) nuclear ribosomal RNA gene, and the minichromosome maintenance complex component 7 (*MCM7*) protein-coding gene. Our phylogenetic reconstruction of *Hesperomyces* based on a concatenated ITS–LSU–*MCM7* dataset revealed *Hesperomyces* sp. ex *Hy. vinciguerrae* as a member of the *He. virescens* species complex distinct from *He. virescens sensu stricto* (s.s.). It also revealed that the *Hesperomyces* sp. ex *Chilocorus bipustulatus* from Algeria is different from *He. virescens* s.s., which is associated with *Chilocorus stigma* from the USA. This suggests that the species of *Hesperomyces* are not solely segregated by host association, but that there is also a biogeographical component involved. Based on these data, we refrained from referring our material from *Hy. vinciguerrae* to *He. hyperaspidis*. Finally, we discuss the usefulness of *MCM7* as a useful marker for species delimitation in *Hesperomyces*.

## Introduction

There are various symbiotic interactions among insects and fungi, ranging from mutualistic, such as those of the fungus-farming leafcutter ants in the genera *Acromyrmex* and *Atta* (Hymenoptera, Formicidae), to pathogenic and parasitic, such as those of *Beauveria* spp. (Ascomycota, Sordariomycetes) and Laboulbeniales (Ascomycota, Laboulbeniomycetes) (Bustamante et al., 2019; Biedermann and Vega, 2020; Haelewaters et al., 2021b). The species of Laboulbeniales are biotrophic microfungi growing externally on the exoskeleton of their arthropod hosts. They are characterized by the formation of multicellular thalli instead of hyphae and a mycelium (Blackwell et al., 2020; Haelewaters et al., 2021a). The study of Laboulbeniales has been neglected by mycologists due to their minute size, insignificant morphological appearance, and inability to grow in axenic culture (Haelewaters et al., 2021b). Recent molecular work has indicated that morphology alone is not sufficient to delimitate species in several genera of Laboulbeniales: *Arthrorhynchus* Kolen. (Haelewaters et al., 2020), *Chitonomyces* Peyr. (Goldmann and Weir, 2012), *Coreomyces* Thaxt. (Sundberg et al., 2018), *Gloeandromyces* Thaxt. (Haelewaters and Pfister, 2019), *Hesperomyces* Thaxt. (Goldmann et al., 2013; Haelewaters et al., 2018), *Laboulbenia* Mont. and C.P. Robin (Haelewaters et al., 2019a), and *Nycteromyces* Thaxt. (Van Caenegem and Haelewaters, unpublished data). In some cases, too many morphological species have been previously recognized. For example, in the genus *Chitonomyces*, 13 morphological species actually represent six phylogenetic species (Goldmann and Weir, 2012). Alternatively, too few species have been recognized in other genera. This pertains to cryptic diversity, with one taxon consisting of several morphologically indistinguishable species. An example of this can be found in the genus *Hesperomyces* (Haelewaters et al., 2018).

The genus *Hesperomyces* was erected by Thaxter (1891) to accommodate *Hesperomyces virescens* Thaxt. on *Chilocorus stigma* (Say, 1835). Since its description, *He. virescens*, in a broad sense, has been reported on more than 30 species of ladybirds (Coleoptera, Coccinellidae) (Haelewaters and De Kesel, 2017). Haelewaters et al. (2018) revealed that *He. virescens* is a complex of species segregated according to host association based on landmark-based geometric, morphometric, ecological, and molecular phylogenetic data. Since then, three species within the complex have been described: *Hesperomyces halyziae* (Haelewaters and De Kesel, 2020), *Hesperomyces harmoniae* (Haelewaters et al., 2022b), and *Hesperomyces parexochomi* (Crous et al., 2021).

In this paper, we present the first molecular data for *He. virescens sensu lato* (s.l.) on *Hyperaspis* and expand our understanding of the diversity within this complex. We also evaluate the usefulness of the minichromosome maintenance complex component 7 (*MCM7*) gene as a secondary barcode for Laboulbeniales.

## Materials and methods

### Collection of insects and morphological study

Ladybirds were collected on the island of Fuerteventura, Canary Islands, Spain. Specimens were shaken down from host plants on a 1-m × 1-m white beating sheet and preserved in 90% ethanol. Identification of the ladybirds was based on morphological and anatomical features, including the type of reproductive organs (Romanowski et al., 2019).

Thalli were removed from the left elytron under a Novex RZB-PL 65.500 dissecting microscope (Novex, Arnheim, Netherlands) at ×10 to ×45 magnification using Minutien pins (#1208SA; BioQuip, Rancho Dominguez, CA, USA) inserted onto wooden rods. Permanent slides were made using the double-coverslip mounting technique as described by Liu et al. (2020), with one modification: the thalli were placed in a droplet of 1:1 Hoyer’s medium/glycerin mixture instead of pure Hoyer’s medium because our Hoyer’s medium dried quickly. Mounted thalli were viewed at ×200 to ×1,000 magnification under an Olympus BH-2 microscope (Olympus, Center Valley, PA, USA). Images were generated with a Nikon DS-Fi3 microscopy camera mounted on an Eclipse Ni-U compound microscope (Nikon, Nelville, NY, USA) equipped with differential interference contrast (DIC) optics and processed using NIS-Elements BR 5.0.03 imaging software (Nikon).

The thalli, cells, structures, and ascospores were measured using ImageJ 1.51h Image Processing and Analysis software (Abramoff et al., 2004). All measurements were taken as described and illustrated by Haelewaters et al. (2018). Measurements in the morphological description were noted as (*a*–)*b*–*c*–*d*(–*e*) [*n*], with *a* and *e* as extreme values, *b* and *d* denote the mean ± standard deviation, *c* represents the mean, and *n* is the number of structures measured. Ladybirds were preserved at the Purdue Entomology Research Collection, West Lafayette, Indiana, USA (PERC), and permanent slides of Laboulbeniales were deposited in the Herbarium Universitatis Gandavensis, Ghent, Belgium (GENT).

### DNA extraction, PCR amplification, and sequencing

DNA extractions were performed using the REPLI-g Single Cell Kit (Qiagen, Stanford, CA, USA). A Minutien pin inserted onto a wooden rod for holdfast was submerged in glycerin to prevent the thalli from flying away during transfer. The thalli were detached from the host and placed in a droplet of glycerin on a microscope slide. To ensure successful lysis, we cut every perithecium transversally once using a no. 10 surgical blade on a disposable Bard-Parker handle (Aspen Surgical, Caledonia, MI, USA). The thalli were then placed in 0.2-ml PCR tubes with 4 µl of phosphate-buffered saline. After the addition of 3 ml of prepared D2 buffer, the tubes were incubated at 65°C for 30 min. Subsequent steps followed the manufacturer’s instructions (Qiagen).

The small subunit (SSU), the internal transcribed region (ITS), and the large subunit (LSU) of the nuclear ribosomal RNA gene were amplified using the following primer pairs: NSL1/NSL2 for SSU (Haelewaters et al., 2015), ITShespL/ITShespR for ITS (Haelewaters et al., 2019b), and LR0R/LR5 for LSU (Vilgalys and Hester, 1990; Hopple, 1994). Additionally, the *MCM7* protein-coding gene was amplified using the primer pair MCM7-709for/MCM7-1384rev (Schmitt et al., 2009). The PCR reactions (25 µl in total) consisted of 13.3 µl of RedExtract Taq polymerase (Sigma-Aldrich, St. Louis, MO, USA), 2.5 µl of each 10 µM primer, 5.45 µl of ddH_2_O, and 1 µl of DNA extract. The PCR cycling conditions used were as follows: for SSU, an initial denaturation at 94°C for 5 min; 39 cycles of denaturation at 94°C for 30 s, annealing at 50°C for 45 s, and extension at 72°C for 90 s; and a final extension at 72°C for 10 min; for ITS, an initial denaturation at 94°C for 3 min; 34 cycles of denaturation at 94°C for 1 min, annealing at 50°C for 45 s, and extension at 72°C for 90 s; and a final extension at 72°C for 10 min; for LSU, an initial denaturation at 94°C for 5 min; 34 cycles of denaturation at 94°C for 30 s, annealing at 50°C for 45 s, and extension at 72°C for 1 min; and a final extension at 72°C for 7 min; for *MCM7*, an initial denaturation at 94°C for 5 min; 10 cycles of denaturation at 94°C for 45 s, annealing at 55°C (−1°C/cycle) for 50 s, and extension at 72°C for 1 min; 24 cycles of denaturation at 94°C for 45 s, annealing at 47°C for 50 s, and extension at 72°C for 1 min; and a final extension at 72°C for 5 min.

The PCR products were purified using 1.5 µl of Exo-FAP (0.5 µl exonuclease I and 1 µl FAST alkaline phosphatase) (Thermo Fisher Scientific, Waltham, MA, USA) per 10 µl of PCR product at 37°C for 15 min, followed by deactivation at 85°C for 15 min. The purified PCR products were sequenced using an automated ABI 3730 XL capillary sequencer (Life Technology at Macrogen, Amsterdam, Netherlands). The forward and reverse sequence reads were assembled and edited in Sequencher version 5.4.6 (Gene Codes Corporation, Ann Arbor, MI, USA). Newly generated sequences were submitted to NCBI GenBank (https://www.ncbi.nlm.nih.gov/genbank/; accession numbers are shown in **Table 1**).

**Table 1:** Details of all the isolates used in the study, including species name, country of collection, host species, and the GenBank accession numbers of the small subunit (SSU), internal transcribed spacer (ITS), large subunit (LSU), and minichromosome maintenance complex component 7 (*MCM7*) sequences.

Species	Isolate	Country	Host	SSU	ITS	LSU	*MCM7*
*Hesperomyces* aff. *coleomegillae*	D. Haelew. 1287b	Panama	*Coleomegilla maculata* (De Geer, 1775)		OL335932	MG745334	
*Hesperomyces* aff. *coleomegillae*	D. Haelew. 1291c	Panama	*Coleomegilla maculata*		OL335933	MG745335	
*Hesperomyces coccinelloides*	D. Haelew. 1428a	Spain	*Stethorus tenerifensis* Fürsch, 1987		OL335930		OP947140
*Hesperomyces coccinelloides*	D. Haelew. 1428b	Spain	*Stethorus tenerifensis*		OL335931	OL335915	OP947141
*Hesperomyces halyziae*	D. Haelew. 955b	Netherlands	*Halyzia sedecimguttata* (Linnaeus, 1758)		MG757813		
*Hesperomyces halyziae*	D. Haelew. 4209a	Netherlands	*Halyzia sedecimguttata*	OP933652	OP933656	OP933659	
*Hesperomyces harmoniae*	D. Haelew. 648c	South Africa	*Harmonia axyridis* (Pallas, 1773)	KU574863	KU574864	KU574865	
*Hesperomyces harmoniae*	D. Haelew. 1174a	Netherlands	*Harmonia axyridis*		MG757815	MG745345	
*Hesperomyces harmoniae*	D. Haelew. 1268b	Japan	*Harmonia axyridis*	MG760610	MG757829	MG745357	OP037811
*Hesperomyces harmoniae*	D. Haelew. 1439a	USA	*Harmonia axyridis*		MN397128	MN397128	OP037812
*Hesperomyces harmoniae*	D. Haelew. 1551b	Czech Republic	*Harmonia axyridis*		OL335935		
*Hesperomyces harmoniae*	D. Haelew. 1808b	USA	*Harmonia axyridis*		OL335936	OL335921	
*Hesperomyces parexochomi*	D. Haelew. 1462a	Spain	*Parexochomus nigripennis* Erichson, 1843		MZ994855		
*Hesperomyces parexochomi*	D. Haelew. 1690d	Spain	*Parexochomus nigripennis*	MZ994884	MZ994863	MZ994874	OP947154
*Hesperomyces parexochomi*	D. Haelew. 1691c	Spain	*Parexochomus nigripennis*	MZ994885	MZ994864	MZ994875	OP947153
*Hesperomyces parexochomi*	D. Haelew. 1465a	Spain	*Parexochomus quadriplagiatus* (Wollaston, 1864)	MZ994881	MZ994860	MZ994871	OP947156
*Hesperomyces parexochomi*	D. Haelew. 1465b	Spain	*Parexochomus quadriplagiatus*		MZ994868	MZ994879	OP947155
*Hesperomyces parexochomi*	D. Haelew. 1584a	Spain	*Parexochomus quadriplagiatus*	MZ994880	MZ994858	MZ994869	
*Hesperomyces virescens* s.l.	D. Haelew. 1193g	Denmark	*Adalia bipunctata* (Linnaeus, 1758)	MG760599	MG757817	MG745346	OP947147
*Hesperomyces virescens* s.l.	D. Haelew. 1199h	Sweden	*Adalia bipunctata*		MG757818	MG745347	
*Hesperomyces virescens* s.l.	D. Haelew. 1231a	Italy	*Adalia bipunctata*		MG757821	MG745350	OP947146
*Hesperomyces virescens* s.l.	D. Haelew. 1248b	Italy	*Adalia decempunctata* (Linnaeus, 1758)	MG760606	MG757823	MG745353	
*Hesperomyces virescens* s.l.	D. Haelew. 1249a	Italy	*Adalia decempunctata*		MG757824		
*Hesperomyces virescens* s.l.	D. Haelew. 655c	South Africa	*Cheilomenes propinqua* (Mulsant, 1850)	KU574866	MG757804	KU574867	
*Hesperomyces virescens* s.l.	D. Haelew. 659b	South Africa	*Cheilomenes propinqua*		MG757805	MG745342	
*Hesperomyces virescens* s.l.	D. Haelew. 1259a	South Africa	*Cheilomenes propinqua*		MG757828		
*Hesperomyces virescens* s.l.	D. Haelew. 924a	Panama	*Cycloneda sanguinea* (Linnaeus, 1763)		MG757808		
*Hesperomyces virescens* s.l.	D. Haelew. 1374a	Panama	*Cycloneda sanguinea*		MG757831		
*Hesperomyces virescens* s.l.	D. Haelew. 3187a	Czech Republic	*Hippodamia tredecimpunctata* (Linnaeus, 1758)		OL335937	OL335923	
*Hesperomyces virescens* s.l.	D. Haelew. 1809c	Chile	*Hippodamia variegata* (Goeze, 1777)			OL335922	
*Hesperomyces virescens* s.l.	D. Haelew. 954e	USA	*Olla v-nigrum* (Mulsant, 1866)		MG757812		OP947148
*Hesperomyces virescens* s.l.	D. Haelew. 1200h	USA	*Olla v-nigrum*	MG760601	MG757819	MG745348	OP947150
*Hesperomyces virescens* s.l.	D. Haelew. 1200i	USA	*Olla v-nigrum*	MG760602	MG757820	MG745349	OP947149
*Hesperomyces virescens* s.l.	D. Haelew. 3202a	Mexico	*Olla v-nigrum*		OL335938	OL335925	
*Hesperomyces virescens* s.l.	JP352b	USA	*Olla v-nigrum*	MG760581	MG757798	MG745337	
*Hesperomyces virescens* s.l.	D. Haelew. 1250b	USA	*Psyllobora vigintimaculata* (Say, 1824)	MG760607	MG757825	MG745354	
*Hesperomyces virescens* s.l.	D. Haelew. 1250c	USA	*Psyllobora vigintimaculata*		MG757826	MG745355	OP947151
*Hesperomyces virescens* s.l.	D. Haelew. 1251b	USA	*Psyllobora vigintimaculata*	MG760609	MG757827	MG745356	OP947152
*Hesperomyces virescens* s.s.	D. Haelew. 1444a	USA	*Chilocorus stigma* (Say, 1835)		MT373697	OL335916	
*Hesperomyces virescens* s.s.	D. Haelew. 1444b	USA	*Chilocorus stigma*		MT373698	OL335917	
*Hesperomyces* sp.	D. Haelew. 4049a	Algeria	*Chilocorus bipustulatus* (Linnaeus, 1758)	OP933651	OP933655	OP933658	
*Hesperomyces* sp.	D. Haelew. 3939b	Spain	*Hyperaspis vinciguerrae* Capra, 1929	OP933653		OP933649	OP947144
*Hesperomyces* sp.	D. Haelew. 3939c	Spain	*Hyperaspis vinciguerrae*	OP933654	OP933657	OP933650	OP947145
*Hesperomyces* sp.	D. Haelew. 928g	Panama	*Azya orbigera* (Mulsant, 1850)	MG760592	MG745343	MG745343	

### Phylogenetic analyses

Newly generated sequences were supplemented by the sequences downloaded from NCBI GenBank, resulting in 19 SSU, 42 ITS, 35 LSU, and 17 *MCM7* sequences (**Table 1**). We used *Hesperomyces* ex *Azya orbigera* Mulsant, 1850, *Hesperomyces coccinelloides* (Thaxt.) Thaxt., and *Hesperomyces coleomegillae* W. Rossi and A. Weir as outgroups (Haelewaters et al., 2022b). We aligned the sequences by locus with the E-INS-i strategy using MAFFT (Multiple Alignment using Fast Fourier Transform) version 7 (Kuraku et al., 2013; Katoh et al., 2019). The sequences were manually trimmed using the BioEdit Sequence Alignment Editor version 7.2.6 (Hall, 1999) and concatenated in SequenceMatrix 1.9 (Vaidya et al., 2011).

We constructed five datasets: each marker individually (ITS, LSU, and *MCM7*) and two concatenated datasets (ITS–LSU and ITS–LSU–*MCM7*). The ITS dataset was partitioned into the ITS1 spacer, the conserved 5.8S gene, and the ITS2 spacer. The concatenated three-locus dataset included five partitions: the ITS1 and ITS2 spacer regions, the 5.8S gene, LSU, and *MCM7*. Likewise, the concatenated two-locus dataset included four partitions (ITS1, 5.8S, ITS2, and LSU). Following previous work in this species complex (Haelewaters et al., 2018; Haelewaters et al., 2022b), we only used a selection of publicly available sequences for the ITS, LSU, and the concatenated datasets. To examine the utility of *MCM7* as a secondary barcode marker, we did use all available (17) *Hesperomyces* sequences in the single-partition dataset of this marker.

Models for nucleotide substitution were selected for each partition using ModelFinder (Kalyaanamoorthy et al., 2017) according to the corrected Akaike information criterion (AICc). Maximum likelihood (ML) analyses were inferred using IQ-TREE (Nguyen et al., 2015) under partitioned models (Chernomor et al., 2016). Ultrafast bootstrapping was performed with 1,000 replicates (Hoang et al., 2018). Phylogenetic trees were visualized in FigTree (http://tree.bio.ed.ac.uk/software/figtree/) and edited using Inkscape (http://www.inkscape.org).

## Results

**Figure 1:**
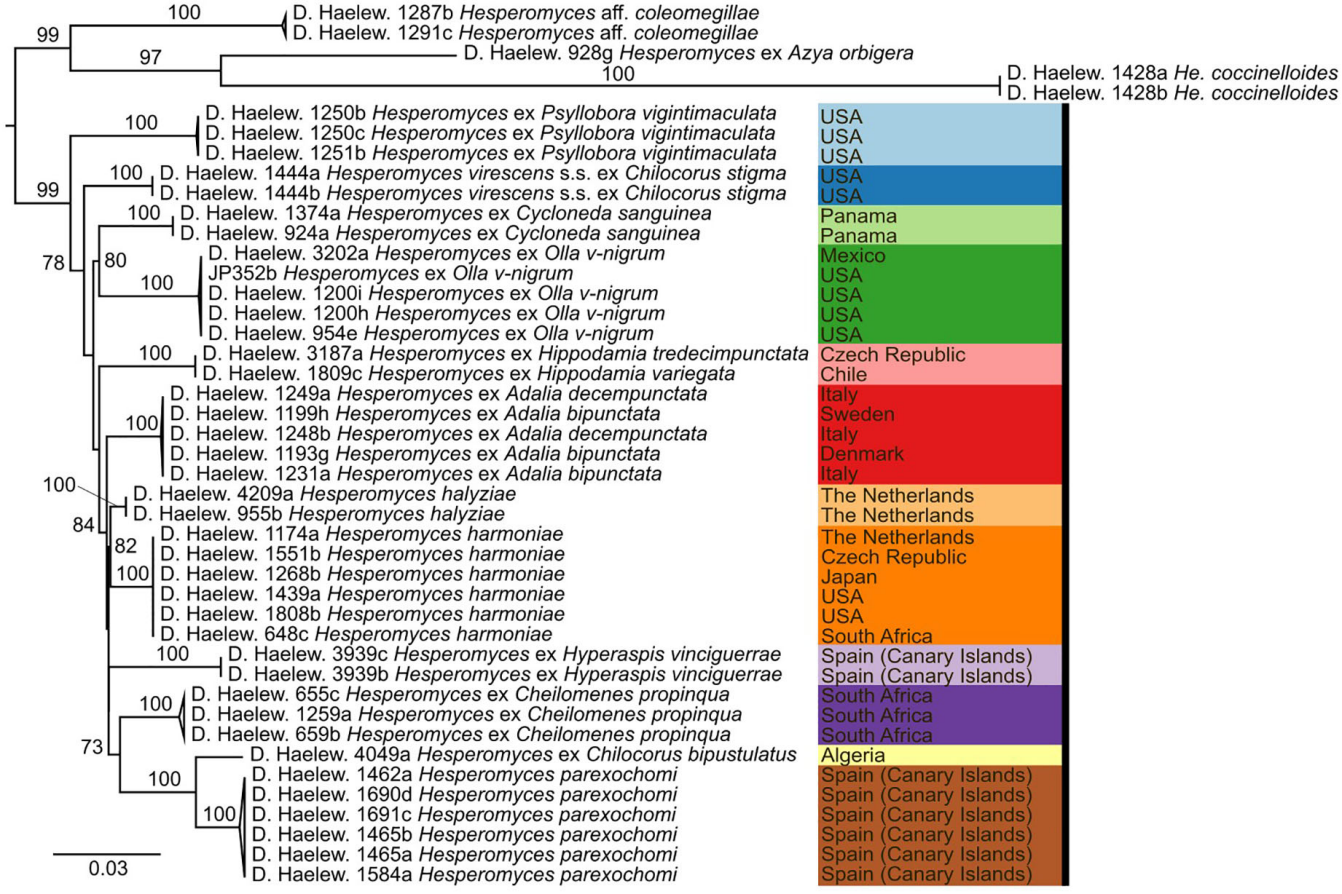
Phylogenetic tree obtained from the maximum likelihood (ML) analysis of a three-locus (ITS–LSU–*MCM7*) dataset. For every node, the ML bootstrap value (≥70) is given above or next to the branch leading to that node. Species within the *Hesperomyces virescens* complex are each indicated with their own color. Color scheme from https://colorbrewer2.org by C.A. Brewer, Geography, Pennsylvania State University.

**Figure 2:**
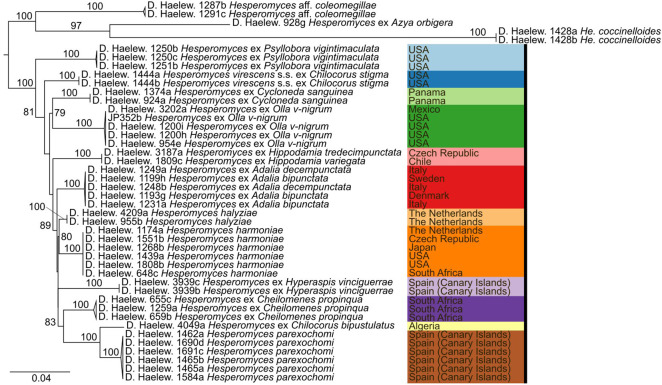
Phylogenetic tree obtained from the maximum likelihood (ML) analysis of a two-locus (ITS–LSU) dataset. For every node, the ML bootstrap value (≥70) is given above or next to the branch leading to that node. Species within the *Hesperomyces virescens* complex are each indicated with their own color, as in **Figure 1**.

**Figure 3:**
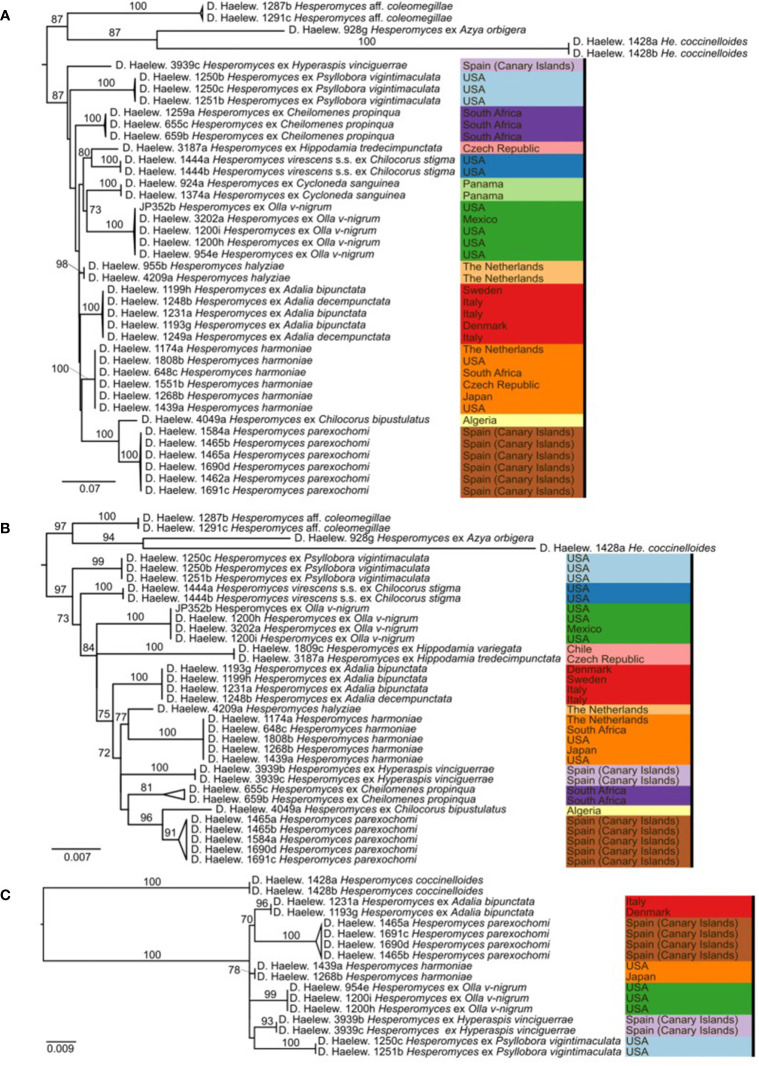
Phylogenies of the *Hesperomyces virescens* species complex, reconstructed from the internal transcribed spacer (ITS) **(A)**, large subunit (LSU) **(B)**, and minichromosome maintenance complex component 7 (*MCM7*) **(C)** datasets. For every node, the ML bootstrap value (≥70) is given above or next to the branch leading to that node. Species within the *He. virescens* complex are each indicated with their own color, as in **Figure 1**.

We generated 26 new sequences for this study (**Table 1**). The concatenated ITS–LSU–*MCM7* dataset consisted of 2,362 characters for 44 isolates. The selected models for each partition in our concatenated dataset were: TPM2+F+G4 (ITS1, 414 bp; −lnL = 4,130.865), TNe (5.8S, 160 bp; −lnL = 579.759), TIM2+F+G4 (ITS2, 272 bp; −lnL = 3,194.845), TIM+F+G4 (LSU, 894 bp; −lnL = 4,549.320), and TIM3e+G4 (*MCM7*, 622 bp; −lnL = 2,790.124). The models for the unpartitioned alignments and the concatenated ITS–LSU dataset were identical. The reconstructed phylogenies of *He. virescens* s.l. are shown in **Figure 1** (for the concatenated ITS–LSU–*MCM7* dataset), **Figure 2** (for the concatenated ITS–LSU dataset), and **Figure 3** (for the ITS, LSU, and *MCM7* datasets). The *He. virescens* s.l. complex consists of 12 lineages, each with maximum support. The SSU sequences are too conserved and cannot be used to delimit species or to identify isolates to the species level (**Supplementary File 1**).

### Taxonomy

*Hesperomyces hyperaspidis* Thaxt., Mem. Am. Acad. Arts Sci., ser. 2 16(1):111 (1931)

#### Material examined

Trinidad and Tobago. Trinidad Island, vicinity of the City of Port of Spain, no date, *leg.* R. Thaxter, on *Hyperaspis* sp. (Coleoptera, Coccinellidae) (no. 2896), slide FH 4989 (holotype at FH, one mature thallus and one broken thallus from elytra); Northern Range hills, slopes of El Tucuche, April 1929, *leg.* P.J. Darlington, on *Hyperaspis* sp. (Coleoptera, Coccinellidae) (no. 3637), slide FH 4990 (FH, one mature and two submature thalli).

#### Hosts and distribution

Described on *Hyperaspis* sp. from Trinidad and reported on *Hyperapsis festiva* Mulsant, 1850, from French Guiana (Bernardi et al., 2014), although only studied based on morphology.

#### Notes

**Figure 4:**
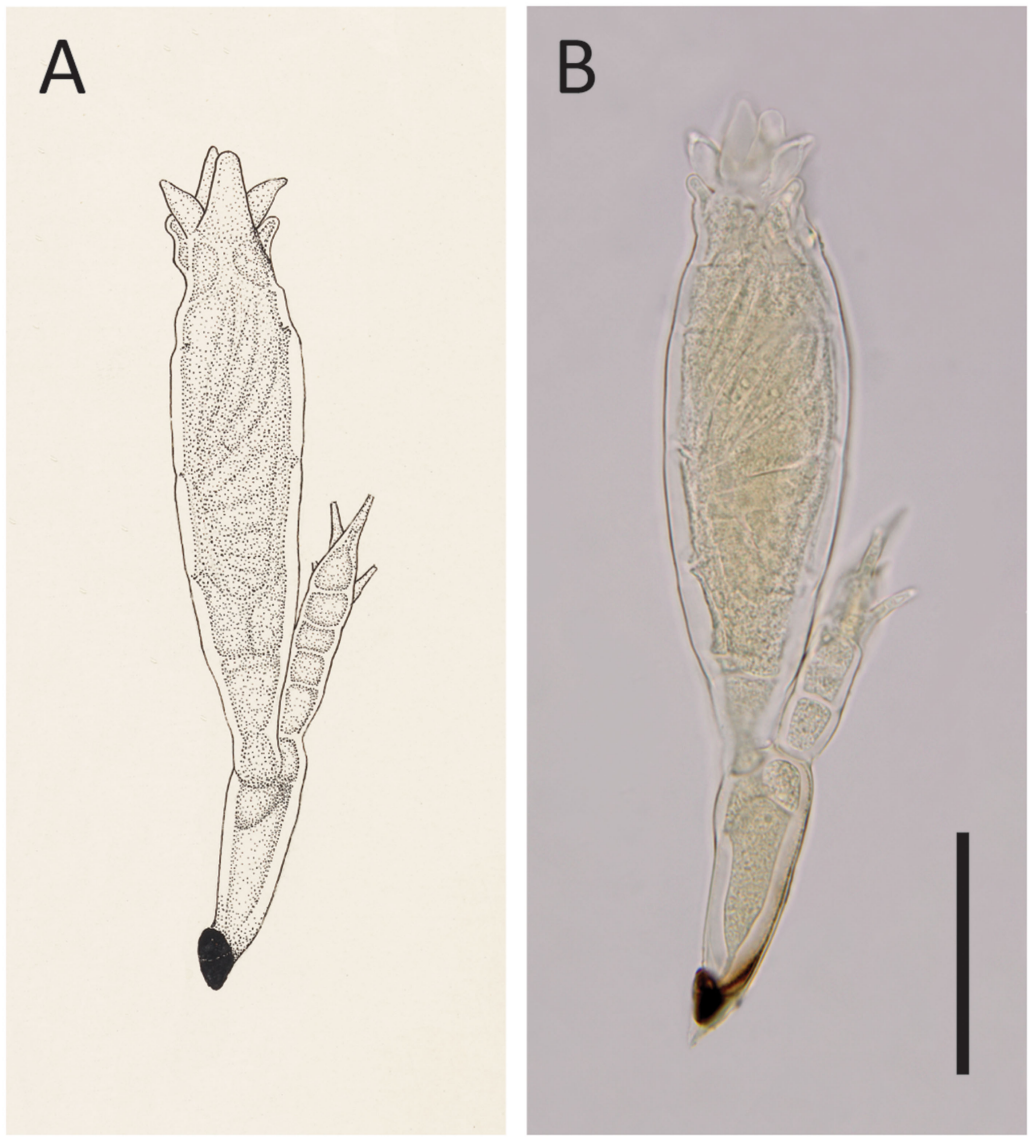
*Hesperomyces hyperaspidis* Thaxt., slide FH 4989 (holotype). **(A)** An enlargement of Thaxter’s (1931) original Plate XX: Figure 22. Courtesy of the Archives of the Farlow Herbarium of Cryptogamic Botany, Harvard University. **(B)** Photo of the same thallus that stood model for Thaxter’s drawing. Scale bar 50 μm.

The description of this species was based on only one mature thallus (Thaxter, 1931); thus, variations in morphology could not be evaluated. According to Thaxter (1931), *He. hyperaspidis* differs from other species of *Hesperomyces* by the length of the six lobes at the perithecial tip (see **Figure 4**). Bernardi et al. (2014) evaluated the usefulness of this morphological characteristic and concluded that the lengths of the lobes were overlapping among species. As a result, these authors synonymized *He. hyperaspidis* with *He. virescens*.

We had access to the protologue material of *He. hyperaspidis*, which comprises the holotype slide (FH 4989) and one uncited slide (FH 4990) made by Thaxter. Our measurements of the single mature thallus in the holotype slide differed from those in the protologue (Thaxter, 1931). Thaxter’s measurements are sometimes at variance with modern work; Haelewaters and Rossi (2015) reported on measurements of thalli that differed from those reported by Thaxter. A comparison of these measurements is given in **Table 2**.

**Table 2:** Measurement of the different structures of the single mature thallus of *Hesperomyces hyperaspidis* in Thaxter’s holotype slide, as described in Thaxter (1931) and re-measured by us.

	Receptacle	Cell VI	Length of appendage	Perithecium	Total length
Thaxter (1931)	55 × 17	21 × 17	62	110 × 25	180
Our measurements	58 × 19	21 × 16	58	119 × 37	193

Measurements are noted as length × width in micrometers, unless stated otherwise.

*Hesperomyces* sp. ex *Hyperaspis vinciguerrae*

#### Material examined

Spain. Canary Islands, Las Palmas, Fuerteventura Island, Jandia, Ventura Shopping Center, 28.053 N, 14.324 W, November 27, 2021, *leg.* Jerzy Romanowski, on *Hy. vinciguerrae* Capra, 1929 (PERC), slide D. Haelew. 3939a (GENT, nine mature thalli from the left elytron), and D. Haelew. 3939d (GENT, 20 mature thalli from the left elytron).

#### Description

The thallus is (174–)200–222–244(–254) μm long from the foot to the perithecial apex; hyaline to yellowish green [29]. The receptacle is (41–)45–48–51(–53) μm long [29]. Cell I is (28–)34–36–38(–39) × (13–)14–15–16(–17) μm, triangular to quadrangular, and is longer than broad [28]. Cell II is (15–)17–18–19(–20) × (9–)10–11–12(–13) μm, longer than broad, and subtrapezoidal [22]. Cell III is (11–)12–13–14(–16) × (8–)9–11–13(–16) μm, shorter than cell II, almost isodiametric, and is dorsally convex [26]. The primary appendage is (38–)43–45–47(–51) μm long, consisting of four superposed cells, with a distinct constricted septum between cell III and the basal cell. The basal cell is (12–)13–14–15(–17) μm long, longer than any of the other cells of the appendage, the remaining cells each bearing one antheridium directed outwardly and the uppermost cell bearing one or two antheridia and a terminal spinous process, which is the original ascospore apex [21]. The antheridia are (13–)16–17–18(–20) μm long, with outwardly straight to curved efferent necks, (6–)7–8–9(–10) μm [22]. Cell VI is (22–)25–29–33(–37) × (15–)16–18–20(–22) μm, rather stout, broadening distally [29]. The perithecium is (120–)134–152–169(–178) × (28–)31–37–43(–47) μm, on average four times longer than broad, asymmetric, fusiform, and broadest near the middle, then gradually tapering toward a short, broad, indistinct neck and an asymmetrical apex. The septa between the horizontal tiers of wall cells are marked by constrictions. The perithecial tip has two lower lobes, two upper lobes, and two prominent lips surrounding the ostiole; lower lobes are minute, while the upper lobes are (10–)11–12–13(–14) μm long, unicellular, thumb-like, usually curved outwards, and their tips not exceeding the perithecial apex. The ostiole has two lips, one lip triangular and the other slightly shorter, rounded [29]. The ascospores are two-celled, (46–)48–53–57(–57) × 4.1–4.6–5.1(–5.3) μm, with a gelatinous sheath covering the larger cell [7].

#### Material sequenced

Spain. Canary Islands, Las Palmas, Fuerteventura Island, Jandia, Ventura Shopping Center, 28.053 N, 14.324 W, November 27, 2021, *leg.* Jerzy Romanowski, on *Hy. vinciguerrae* (PERC), isolate D. Haelew. 3939b (six mature thalli, left elytron; SSU = OP933653, LSU = OP933649, *MCM7* = OP947144); *ibid.*, isolate D. Haelew. 3939c (five mature thalli, left elytron; SSU = OP933654, ITS = OP933657, LSU = OP933650, *MCM7* = OP947145).

#### Hosts and distribution

Reported on *Hy. vinciguerrae* Capra, 1929, from Spain (this paper).

#### Notes

**Figure 5:**
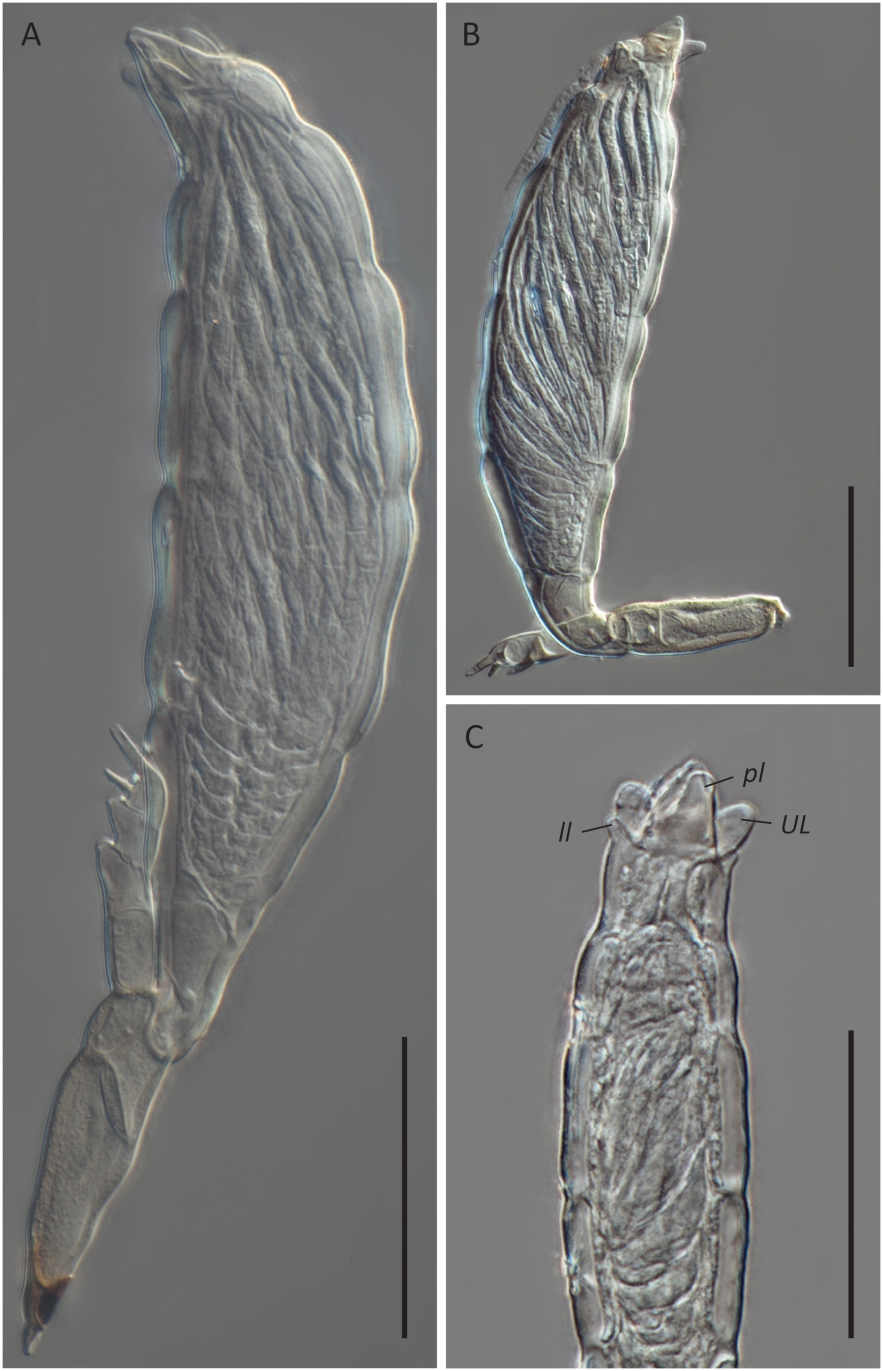
*Hesperomyces* sp. ex *Hyperaspis vinciguerrae*. **(A)** Mature thallus, slide D. Haelew. 3939a. **(B)** Mature thallus, slide D. Haelew. 3939a. Cell VI is highly bent. **(C)** Perithecial tip of a mature thallus, slide D. Haelew. 3939d. Indicated are the two lower lobes (*ll*), the two upper lobes (*UL*), and the two perithecial lips (*pl*), similar to *Hesperomyces hyperaspidis*. Scale bar 50 μm.

*Hesperomyces* sp. ex *Hy. vinciguerrae* is part of the near-cryptic *He. virescens* species complex (Haelewaters et al., 2018). Morphologically, it is very similar to the other species in this complex (**Figure 5**). Given that our material was associated with a *Hyperaspis* host, we compared the morphological characteristics with those of *He. hyperaspidis*. The thalli from our collection had short lobes at the tip of the perithecium, similar to those of *He. hyperaspidis* described by Thaxter (1931). Most of the thalli were bent in cell VI, mostly at a 90° angle toward anterior. This is different from the available material of *He. hyperaspidis*, but this difference could be attributed to morphological plasticity. The receptacle of the thallus in the holotype of *He. hyperaspidis* is 10 µm longer than the mean length of the receptacle we measured in our material. The total length of the thalli we studied is, on average, 30 µm longer than the holotype of *He. hyperaspidis*.

*Hesperomyces* sp. ex *Hy. vinciguerrae* formed a distinct species-level clade supported by ITS, LSU, and *MCM7* data. Unique molecular autapomorphies and motifs in the ITS were found at positions 130–132 (5'-TTC-3') (insertion), 140 (C), 148 (A), 179 (G), 197 (C), 206 (G), 232 (G), 250 (G), 343 (G), 391 (C), 403 (T), 407 (T), 597 (T), 658 (T), 682 (C), 683 (A), 724 (G), 742 (A), 818 (G), and 827 (G); in the LSU at positions 116 (G), 173 (C) (insertion), 176 (A), 416 (T), 436 (C), 480 (C), and 490 (A); and in *MCM7* at positions 126 (A), 288 (T), 504 (A), and 528 (A).

Using the NCBI BLAST tool, we searched for sequences with the highest similarity to our newly generated ITS and LSU sequences of *Hesperomyces* sp. ex *Hy. vinciguerrae*. For the ITS sequence, these include: *He. halyziae* [isolate D. Haelew. 955b, GenBank MG757813; identity = 717/765 (94%), 6 (0%) gaps]; *He. harmoniae* [isolate D. Haelew. 1439a, GenBank MN397128; identity = 720/772 (93%), 8 (1%) gaps]; *He. harmoniae* [isolate D. Haelew. 1268b, GenBank MG757829; identity = 720/772 (93%), 8 (1%) gaps]; *He. harmoniae* [isolate D. Haelew. 943b, GenBank MG757810; identity = 720/772 (93%), 8 (1%) gaps]; and *He. harmoniae* [isolate D. Haelew. 1808b, GenBank OL335936; identity = 720/772 (93%), 8 (1%) gaps]. For the LSU sequences, these are: *He. virescens* ex *Adalia bipunctata* [isolate D. Haelew. 1232a, GenBank MG745351; identity = 882/899 (98%), 1 (0%) gap]; *He. virescens* ex *A. bipunctata* [isolate D. Haelew. 1199h, GenBank MG745347; identity = 882/899 (98%), 1 (0%) gap]; *He. virescens* ex *A. bipunctata* [isolate D. Haelew. 1231a, GenBank MG745350; identity = 882/900 (98%), 2 (0%) gaps]; *He. virescens* ex *A. bipunctata* [isolate D. Haelew. 1231a, GenBank MG745350; identity = 882/900 (98%), 2 (0%) gaps]; *He. parexochomi* [isolate D. Haelew. 1584a, GenBank MZ994870; identity = 882/900 (98%), 2 (0%) gaps]; and *He. parexochomi* [isolate D. Haelew. 1693a, GenBank MZ994870; identity = 881/899 (98%), 1 (0%) gap].

## Discussion

### *MCM7* as a secondary marker in Laboulbeniales

The ITS region has been proposed as a universal barcode for fungi (Schoch et al., 2012). General fungal ITS primers have been used in a few molecular studies to successfully amplify the ITS region to delimit the species of Laboulbeniales (e.g., Goldmann and Weir, 2012; Goldmann et al., 2013; Sundberg et al., 2018). However, the amplification success of the ITS region substantially differed among the taxa of Laboulbeniales when using general fungal primers such as ITS1f, ITS9mun, and ITS4 (Haelewaters et al., 2015; Haelewaters et al., 2018; Walker et al., 2018). A new primer pair, ITSHespL/ITSHespR, with improved specificity for *Hesperomyces* was described (Haelewaters et al., 2018). Since this design, we have successfully used these specific primers to amplify the ITS region of *Hesperomyces*, including in this study.

Recent studies have shown that LSU, in contrast to ITS, is easier to amplify using general fungal primer pairs such as LR0R/LR5, LIC24R/LR3, and NL1/NL4 (Haelewaters et al., 2015; Haelewaters et al., 2018; Walker et al., 2018). Haelewaters et al. (2018) reported that both the ITS and LSU datasets resulted in high support for the species-level clades within *He. virescens* s.l. and suggested further investigation of LSU as a secondary barcode in Laboulbeniales. Thus far, LSU has been used in several studies delimiting species within *Arthrorhynchus*, *Coreomyces*, *Gloeandromyces*, *Hesperomyces*, and *Laboulbenia* (Haelewaters et al., 2018; Sundberg et al., 2018; Haelewaters and Pfister, 2019; Haelewaters et al., 2020; Haelewaters and De Kesel, 2020; Liu et al., 2020).

Liu et al. (2020) generated the first sequences of the translation elongation factor 1α (*TEF1*) for *Gloeandromyces* and *Nycteromyces* species. The phylogeny based on these *TEF1* sequences matched the one based on the LSU sequences, with high support for the species clades. To date, no other studies have used *TEF1* for species delimitation in Laboulbeniales. We continued to test the amplification success of the *TEF1* gene in different genera of Laboulbeniales and have generated sequences for isolates of *Arthrorhynchus* (seven sequences), *Herpomyces* Thaxt. (one sequence), *Hesperomyces* (10 sequences), and *Laboulbenia* (one sequence) (Haelewaters, unpublished data).

In this study, we expanded on the first efforts by Haelewaters et al. (2022b) to generate the *MCM7* sequences of *Hesperomyces* species. We generated 15 new *MCM7* sequences of the *Hesperomyces* species using the primer pair MCM7-709for/MCM7-1384rev and the two-step touchdown PCR protocol indicated above. The amplification of *MCM7* proceeded without issue for all extracts of *Hesperomyces* in the trial. We also successfully amplified the *MCM7* gene for the isolates of *Appendiculina* (one sequence), *Corethromyces* (one sequence), *Herpomyces* (three sequences), *Laboulbenia* (five sequences), and *Nycteromyces* (eight sequences) under the same cycling conditions (Van Caenegem and Haelewaters, unpublished data).

Here, we presented the first phylogeny of *Hesperomyces* based on *MCM7*. The dataset included *Hesperomyces* isolates removed from six host species. All isolates were resolved in six monophyletic clades, and each of the tip nodes (species) was highly to maximally supported in the *MCM7*-based phylogeny (**Figure 3C**), similar to what Haelewaters et al. (2018) reported for their phylogenetic reconstructions based on ITS and LSU. Based on available data, we can only compare ITS, LSU, and *MCM7* for their efficacy to delimit species in *Hesperomyces*. Support for tip nodes was comparably high in all three markers. Conversely, neither marker has the discriminative power to resolve deeper nodes within the complex—but that was not the goal of this study. Given the difficulty to amplify the ITS region, we suggest exploring the utility of *MCM7* as a secondary marker in other genera of the thallus-forming Laboulbeniomycetes. We conclude with the suggestion that *MCM7* is a useful region for the delimitation and identification of *Hesperomyces* species.

### Species of *Hesperomyces* are segregated by host association and geography

All species-level lineages within *He. virescens* s.l. received maximum support in our multilocus phylogeny (ITS–LSU–*MCM7*). The species within this complex are morphologically very similar. However, based on our observations, it seems that the thalli and ascospores of *Hesperomyces* sp. ex *Hy. vinciguerrae* (thallus, 174–254 µm; ascospores, 46–57 µm) are much smaller than those of *He. halyziae* (thallus, 335–453 µm; ascospores, 70–85 µm), *He. harmoniae* (thallus, 290–653 µm; ascospores, 66–106 µm), and *He. parexochomi* (thallus, 251–441 µm; ascospores, 59–74 µm) (Haelewaters and De Kesel, 2020; Crous et al., 2021; Haelewaters et al., 2022b).

The one-host-one-parasite (1H1P) model states that species of Laboulbeniales with a haustorium, like those in the genus *Hesperomyces*, have higher host specificity compared to species without a haustorium because the fungi are in closer contact with their host (Haelewaters et al., 2022a). The species within *He. virescens* s.l. are seemingly segregated by host genus. Support is provided by *He. virescens* s.l. isolates removed from *Adalia*, *Hippodamia*, and *Parexochomus* spp. An undescribed species of *Hesperomyces* has been found on *Adalia bipunctata* (Linnaeus, 1758) and *A. decempunctata* (Linnaeus, 1758) in Denmark, Italy, and Sweden (Haelewaters et al., 2018). Another undescribed species of *Hesperomyces* has been found on both *Hippodamia variegata* (Goeze, 1777) from Chile and *Hippodamia tredecimpunctata* (Linnaeus, 1758) from the Czech Republic. Finally, *He. parexochomi* from the Canary Islands is found on two host species: *Parexochomus nigripennis* Erichson, 1843, and *Parexochomus quadriplagiatus* (Wollaston, 1864) (Crous et al., 2021). In addition, *He. harmoniae* is found almost all over the world where its host, *Harmonia axyridis* (Pallas, 1773), has been introduced, and there have been some observations of *Hesperomyces*-infected *Ha. quadripunctata* (Pontoppidan, 1763), although no isolates are available to confirm their identity (Haelewaters et al., 2022b).

In an earlier version of this paper, we planned to reinstate *He. hyperaspidis* since we thought our thalli from *Hy. vinciguerrae* belonged to this species. However, during the review process, we generated the sequences of *Hesperomyces* sp. ex *Chilocorus bipustulatus* from Algeria. This is an interesting case, as species within the *He. virescens* complex are thought to be segregated by their host genus association and *He. virescens sensu stricto* (s.s.) is described from *Ch. stigma* from the USA (Thaxter, 1891; Haelewaters et al., 2018; Haelewaters et al., 2022b). To our surprise, *Hesperomyces* sp. ex *Ch. bipustulatus* does not form a monophyletic clade with *He. virescens* s.s. ex *Ch. stigma* and is retrieved sister to *He. parexochomi* in both the ITS and LSU phylogenies with maximum support. This result reshaped our thinking on the diversification within the *He. virescens* complex: it appears that species are not simply segregated by their host genus association and that there is a geographical component involved as well.

The host of the holotype material of *He. hyperaspidis*, an unidentified species of *Hyperaspis*, was collected in Trinidad. The infected *Hy. vinciguerrae* in this study was collected in the Canary Islands. To avoid future taxonomic issues, we refrained from 1) assigning our material of *Hesperomyces* sp. ex *Hy. vinciguerrae* to *He. hyperaspidis*; 2) reinstating said species; and 3) formally describing *Hesperomyces* sp. ex *Hy. vinciguerrae* as a new species. However, given all the available knowledge, it stands without a doubt that *He. hyperaspidis* should not be considered a synonym of *He. virescens* s.s., in contrast to the conclusion of Bernardi et al. (2014) based on morphology. Moving forward, *Hyperaspis* ladybirds should be collected in Trinidad and other locations around the world to screen for *Hesperomyces* thalli, extract DNA, and compare the sequences with the recent material from the Canary Islands. We also refrained from describing *Hesperomyces* sp. ex *Ch. bipustulatus* because we found only juvenile thalli.

## Conclusion

Thalli of *Hesperomyces* from a *Hy. vinciguerrae* ladybird, recently collected in the Canary Islands, triggered an integrative taxonomy investigation into the identify of this fungus. Based on the phylogenies inferred from single-locus and multilocus ITS, LSU, and *MCM7* data, we found that the thalli on *Hy. vinciguerrae* belong to another species than *He. virescens* s.s., as do the thalli on *Ch. bipustulatus* from Algeria. The hypothesis that species within the *He. virescens* complex are segregated by their host association appears to be complicated by geography. Thus, we have not assigned *Hesperomyces* sp. ex *Hy. vinciguerrae* to *He. hyperaspidis*, which is described from an unidentified *Hyperaspis* ladybird collected in Trinidad. However, there is good reason to consider *He. hyperaspidis* as a unique species separate from *He. virescens* s.s.

## Data availability statement

All final alignments and unedited trees are available through GitHub: https://github.com/dannyhaelewaters/teamlaboul/tree/main/hesperomyces_hyperaspidis_paper. Newly generated sequences were submitted to the National Center for Biotechnology Information (NCBI) GenBank database (https://www.ncbi.nlm.nih.gov/genbank/), under the following accession numbers: OP933649–OP933659, OP947140–OP947141, and OP9471444–OP947156.

## Author contributions

WVC and DH designed the study. WVC, PC, JR, and DHP collected the data. WVC performed data analysis. DH and JR acquired funding. WVC drafted the manuscript. WVC, DHP, and DH revised the manuscript. All authors contributed to the article and approved the submitted version.

## Supplementary material

Supplementary File 1 Phylogeny of the *Hesperomyces virescens* species complex, reconstructed from the SSU dataset. No ML bootstrap value was ≥70. Species within the *Hesperomyces virescens* complex are each indicated with their own color, as in **Figure 1**.
