# Supplementary figures and images for: Hesperomyces (Fungi, Ascomycota) associated with Hyperaspis ladybirds (Coleoptera, Coccinellidae): Rethinking host specificity
Source: Front Fungal Biol. 2023 Jan 9;3:1040102. doi: 10.3389/ffunb.2022.1040102 (PMC10512334; doi:10.3389/ffunb.2022.1040102)

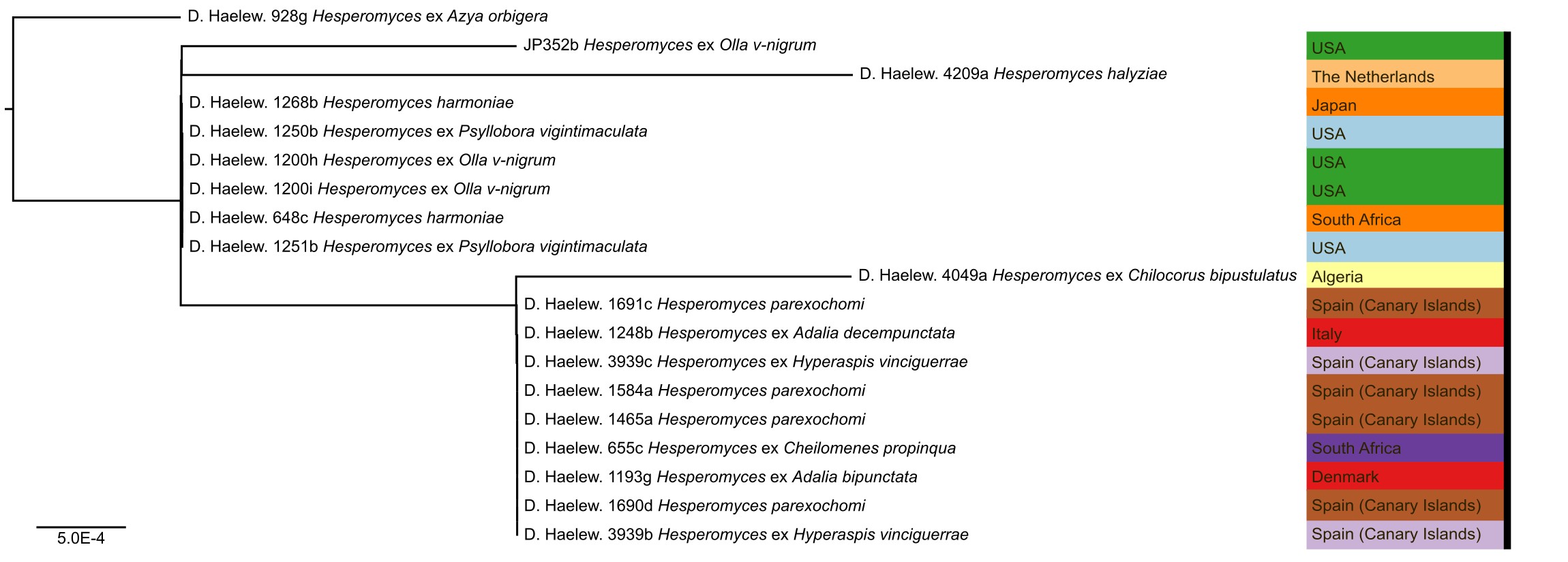

Supplement: Supplementary File 1 — Phylogeny of the Hesperomyces virescens species complex, reconstructed from the SSU dataset. No ML bootstrap value was ≥70. Species within the Hesperomyces virescens complex are each indicated with their own color, as in Figure 1 . [file Image_1.jpeg]
